# Supplementary material for: A two-gene marker for the two-tiered innate immune response in COVID-19 patients
Source: PLoS One. 2023 Jan 17;18(1):e0280392. doi: 10.1371/journal.pone.0280392 (PMC9844909; doi:10.1371/journal.pone.0280392)
Supplement: Attachment — Submitted filename: Comments D-22-29608.docx [file pone.0280392.s001.docx]

Comments

1. The found the MS very fascinating and well organized.
2. The biomarkers you discussed “IFI27& S100A12” are novel regarding COVID-19 severity? but you only discussed it in symptomatic subjects is it possible? if you also take sample of asymptomatic individuals and compare the activation level of both population.
3. Rephrase line 85-92.
