# Supplementary material for: A two-gene marker for the two-tiered innate immune response in COVID-19 patients
Source: PLoS One. 2023 Jan 17;18(1):e0280392. doi: 10.1371/journal.pone.0280392 (PMC9844909; doi:10.1371/journal.pone.0280392)
Supplement: Attachment — Submitted filename: Dr Marwa comments.docx [file pone.0280392.s002.docx]

**Comments to the editor for evaluating the following manuscript**

**Toward routine monitoring of the two-tiered innate immune response in COVID-19 patients**

The authors addressed the two-tiered innate immune response has not been examined in COVID-19.

It is greatly suggested that the manuscript is not ready to be accepted now. I have several comments listed below.

- The tile is not specified for the findings of the study. It must be rewritten to include also the criteria tested.
- The aim of the work in the abstract should be cleared.
- Introduction must be focus on the problem the research dealt with and how will the authors solve this problem in addition to determining the gap in this point in previous researches.
- Is the author depending on databases only for its study?
- Are these databases sufficient for analysis?
- Did the geographical distribution have a role in the data analysis?
- What is the impact of the patient’s age on the severity of the disease and the expression of two-tiered innate immune response?
- The discussion must include the causes for the similarities or differences for all items not only mention what are different or similar than other researches.
- What is the author interpretation if there is any mixed infection in the patients analyzed?
- The author must add a brief conclusion for his study.
- Where is the statistical analysis for this study?

**Typo issues:**

- There were some errors in the structure of several sentences.
- Under all figures, all abbreviations must be mentioned as footnotes.
- The authors must write what each abbreviated word stands for before using the abbreviation for the first time.

**Finally, I cannot accept publishing of this article unless all corrections are made. Therefore, the manuscript is accepted for publication after major revisions**
