# Supplementary material for: A two-gene marker for the two-tiered innate immune response in COVID-19 patients
Source: PLoS One. 2023 Jan 17;18(1):e0280392. doi: 10.1371/journal.pone.0280392 (PMC9844909; doi:10.1371/journal.pone.0280392)
Supplement: Attachment — Submitted filename: Response letter.docx [file pone.0280392.s003.docx]

Response letter

Reviewer #1: Comments
1. I found the MS very fascinating and well organized.

**I appreciate the encouragement from the reviewer.**

2. The biomarkers you discussed “IFI27& S100A12” are novel regarding COVID-19 severity. but you only discussed it in symptomatic subjects. is it possible if you also take sample of asymptomatic individuals and compare the activation level of both population.

**To our knowledge, both the concept of two-tiered innate immune response and the two-gene marker panel are novel for COVID-19. As for the asymptomatic patients, the main aim of this study is to find convenient and efficient markers to monitor the innate immune response in hospitalized COVID-19 patients. Since asymptomatic patients rarely go to hospitals, they can not be monitored by medical professionals anyway. For the same reason, asymptomatic patients are not the focus of many studies. Nevertheless, asymptomatic or non-hospitalized patients were included in a few datasets analyzed in this study. But it’s uncertain how informative it is. Ideally, we would like to have reasonable sample size for asymptomatic and symptomatic patients as well as healthy controls in the same dataset. This statement has been added to the Discussion section.**

3. Rephrase line 85-92.

**The entire paragraph has been revised.**

Reviewer #2: Comments to the author for evaluating the following manuscript
Toward routine monitoring of the two-tiered innate immune response in COVID-19 patients
The authors addressed the two-tiered innate immune response has not been examined in COVID-19.
It is greatly suggested that the manuscript is not ready to be accepted now. I have several comments listed below.
• The tile is not specified for the findings of the study. It must be rewritten to include also the criteria tested.

**Thanks! The title has been rephrased.**

• The aim of the work in the abstract should be cleared.

**The aim of the work has been added to the beginning of the Abstract.**

• Introduction must be focus on the problem the research dealt with and how will the authors solve this problem in addition to determining the gap in this point in previous researches.

**Sorry, the aim of this study was not clearly stated in the previous version of Abstract. It has been revised. The Introduction section has also been revised accordingly.**

• Is the author depending on databases only for its study?

**Yes, this study is based on reanalysis of public datasets.**

• Are these databases sufficient for analysis?

**This study included 1421 samples from 17 independent datasets generated around the globe. The two-tiered innate immune response and the activation pattern of the two marker genes are well reproduced across so many independent datasets. Therefore, the results are quite robust.**

• Did the geographical distribution have a role in the data analysis?

**The samples were collected from around the globe, including Europe, North America, Asia and Australia. The patterns were consistent in different datasets.**

• What is the impact of the patient’s age on the severity of the disease and the expression of two-tiered innate immune response?

**Based on our previous works, age does not have an effect on the expression of IFI27 or S100A12. But it’s a well-known fact that age is a major contributing factor to COVID-19 severity. This statement has been added to the Discussion section in the revised manuscript.**

• The discussion must include the causes for the similarities or differences for all items not only mention what are different or similar than other researches.

**The Discussion section has been revised accordingly.**

• What is the author interpretation if there is any mixed infection in the patients analyzed?

**According to previous studies, bacterial co-infection in COVID-19 patients is not as common as expected earlier (<5%). Therefore, it’s unlikely to have a major effect on the analysis. A comparison of COVID-19 patients with or without coinfection is presented in Figure 9A.**

• The author must add a brief conclusion for his study.

**A Conclusion section has been added to the revised manuscript.**

• Where is the statistical analysis for this study?
**For datasets with sufficiently large sample, p-values for group comparison have been calculated and added to the revised manuscript.**

Typo issues:
• There were some errors in the structure of several sentences.

**The manuscript has been extensively revised to avoid errors.**

• Under all figures, all abbreviations must be mentioned as footnotes.

**This issue regarding figure captions has been taken care of.**

• The authors must write what each abbreviated word stands for before using the abbreviation for the first time.
**The issue regarding abbreviations has been taken care of.**

Finally, I cannot accept publishing of this article unless all corrections are made. Therefore, the manuscript is accepted for publication after major revisions
