# Supplementary material for: A two-gene marker for the two-tiered innate immune response in COVID-19 patients
Source: PLoS One. 2023 Jan 17;18(1):e0280392. doi: 10.1371/journal.pone.0280392 (PMC9844909; doi:10.1371/journal.pone.0280392)
Supplement: Attachment — Submitted filename: Response letter-Rev2.docx [file pone.0280392.s004.docx]

Response letter

Reviewer #1: Comments:
1. The author is suggested to revise the manuscript, and thoroughly read for English correction.

**Thanks! The whole manuscript has been checked and revised.**

Reviewer #2: Comments for evaluating the following manuscript
A two-gene marker for the two-tiered 1 innate immune response in COVID-19 patients
The authors addressed the two-tiered innate immune response has not been examined in COVID-19.
I would like to thank the author for revision, but I have some comments listed below.
• The goal must be written at the end of the introduction section and the authors must arrange the ideas in introduction serially to introduce the problem and how to solve. Moreover, the introduction must not have detailed materials and methods.
**Thanks! The last paragraph of the Introduction section has been revised.**

• The author should illustrate the role of geographical distribution on his results.
**The role of geographic distribution has been added to the Discussion.**

• The discussion needs to be improved regarding the causes for the similarities or differences for majority of the items.
**The Discussion section has been further revised.**

• Under all figures, there were many abbreviations those must be mentioned as footnotes.

**The figure legends have been further revised for clarity.**
